# On the hypothesis-free testing of metabolite ratios in genome-wide and metabolome-wide association studies

**DOI:** 10.1186/1471-2105-13-120

**Published:** 2012-06-06

**Authors:** Ann-Kristin Petersen, Jan Krumsiek, Brigitte Wägele, Fabian J Theis, H-Erich Wichmann, Christian Gieger, Karsten Suhre

**Affiliations:** 1 Institute of Genetic Epidemiology, Helmholtz Zentrum München, Neuherberg, Germany; 2 Institute of Bioinformatics and Systems Biology, Helmholtz Zentrum München, Neuherberg, Germany; 3 Department of Genome-oriented Bioinformatics, Life and Food Science Center Weihenstephan, Technische Universität München, Freising, Germany; 4 Institute of Epidemiology I, Helmholtz Zentrum München, Neuherberg, Germany; 5 Institute of Medical Informatics, Biometry and Epidemiology, Chair of Epidemiology, Ludwig-Maximilians-Universität, München, Germany; 6 Klinikum Grosshadern, Munich, Germany; 7 Faculty of Biology, Ludwig-Maximilians-Universität, Planegg-Martinsried, Germany; 8 Department of Physiology and Biophysics, Weill Cornell Medical College in Qatar, Education City - Qatar Foundation, Doha, Qatar

**Keywords:** p-gain, Metabolomics, MWAS, GWAS, Genome-wide association studies, Metabolome-wide association studies

## Abstract

**Background:**

Genome-wide association studies (GWAS) with metabolic traits and metabolome-wide association studies (MWAS) with traits of biomedical relevance are powerful tools to identify the contribution of genetic, environmental and lifestyle factors to the etiology of complex diseases. Hypothesis-free testing of ratios between all possible metabolite pairs in GWAS and MWAS has proven to be an innovative approach in the discovery of new biologically meaningful associations. The p-gain statistic was introduced as an ad-hoc measure to determine whether a ratio between two metabolite concentrations carries more information than the two corresponding metabolite concentrations alone. So far, only a rule of thumb was applied to determine the significance of the p-gain.

**Results:**

Here we explore the statistical properties of the p-gain through simulation of its density and by sampling of experimental data. We derive critical values of the p-gain for different levels of correlation between metabolite pairs and show that B/(2*α) is a conservative critical value for the p-gain, where α is the level of significance and B the number of tested metabolite pairs.

**Conclusions:**

We show that the p-gain is a well defined measure that can be used to identify statistically significant metabolite ratios in association studies and provide a conservative significance cut-off for the p-gain for use in future association studies with metabolic traits.

## Background

With the advent of modern metabolomics techniques, hundreds of endogenous organic compounds (metabolites) from tissue samples, cell cultures and body fluids can now be measured in a highly standardized and often non-targeted manner. Current technologies are based on liquid chromatography–mass spectrometry (LC-MS), gas chromatography–mass spectrometry (GC-MS), flow injection analysis mass spectrometry (FIA-MS/MS) or nuclear magnetic resonance spectroscopy (NMR) [1-3]. Genome-wide association studies (GWAS) with large numbers of metabolic traits and metabolome-wide association studies (MWAS) with a wide range of biomedical relevant traits are enabled by the newly achieved high-throughput metabolomics capabilities.

Specific ratios between selected pairs of metabolite concentrations (metabolite ratios) have been introduced in the past as biomarkers in many biomedical applications. For instance, medium-chain acyl-CoA dehydrogenase deficiency (MCADD) is detected in systematic “newborn screens” on the basis of elevated blood concentrations of octanoylcarnitine (C8) and other acylcarnitines, in combination with ratios between acylcarnitine concentrations, including hexanoylcarnitine (C6), decanoylcarnitine (C10), decenoylcarnitine (C10:1), C8/C6, C8/C10, and C8/C12 (dodecanoylcarnitine) [4]. The ratio between blood phenylalanine to tyrosine concentrations is used to identify heterozygous carriers of phenylketonuria (PKU) risk alleles [5]. Metabolite ratios are also used as biomarkers to detect specific exposures. For instance, the urinary hydroxyproline to creatinine ratio was proposed as an indicator for personal nitrogen dioxide (NO_2_) exposure [6].

With modern high-throughput technologies, the concept of metabolite ratio analysis has been scaled up to systematically analyzing all possible combinations of ratios between metabolite pairs in a hypothesis-free approach. A number of recently published papers highlight the power of this approach: Altmaier *et al.*[7] applied hypothesis-free metabolite ratio analysis to pre-clinical drug testing in diabetic mice. They linked ratios between sphingolipids that differ by two carbon moieties to a modified beta-oxidation and ratios between different classes of phospholipids to modified activity of enzymes in the phospholipid pathways. In a metabolite association study with smoking, Wang-Sattler *et al.*[8] identified an association with ratios between ester- and ether-bond phospholipids. The biochemical properties of these phospholipids allowed pinpointing the association to the enzymatic action of alkylglycerone phosphate synthase. Using similar approaches, Altmaier *et al.*[9] identified biochemically relevant associations between metabolite ratios and self-reported nutrition habits, and Suhre *et al.*[10] used metabolite ratios to identify functional biomarkers for pre-clinical drug testing of FABP4 inhibitors. Gieger *et al.*[11], Illig *et al.*[12] and Suhre *et al*. [13] introduced hypothesis-free testing of metabolite ratios to GWAS. They showed that using ratios can increase the power of GWAS by tens of orders of magnitude. The leading metabolic traits in 14 out of 15 genetic associations reported by Illig *et al.*[12] and 20 out 37 associations by Suhre *et al*. [13] are ratios between metabolite concentrations (Table 1). Most interestingly, they found that the biochemical nature of the associating metabolite pairs was in nearly all cases directly related to the biochemical function of an enzyme or transporter gene that was encoded at the respective loci. 

**Table 1:** Selected examples of published associations with hypothesis-free testing of metabolite ratios

**Metabolite ratio**	**Association**	**Interpretation**	**Reference**
SM(OH)C28:0/SM(OH)C26:0	Diabetic (db/db) versus wild type mice	Increased beta-oxidation in diabetic mice	Altmaier *et al*., Endocrinology, 2008
PC aa C36:3/PC aa C36:4	*FADS1* genotype	Genetic variance in delta-5 fatty acid desaturation	Gieger *et al.*, PLoS Genetics, 2008
PC aa Cx:y/PC ae Cx:y	Smoking	Reduced or lack of activity of the enzyme alkyl-DHAP in smokers	Wang-Sattler *et al.*, PLoS One, 2008
PC aa C40:3/PC aa C42:5	*ELOVL2* genotype	Genetic variance in elongation of fatty acids	Illig *et al*., Nature Genetics, 2010
Medium chain fatty acids / long chain fatty acids	Diabetes state	Perturbed lipid metabolism associated with diabetes	Suhre *et al*., PLoS One, 2010
PC aa C40:5/PC aa C40:6	Self-reported nutritional intake of polyunsaturated fatty acids	Confirmation of questionnaire based life-style parameters	Altmaier *et al*., Eur. J. Endocrinology, 2011
Ratios between phospholipids with lipid side chains from the C16:0, C16:1, C18:0, C18:1 pool and C20:3, C20:4, C22:4 PUFAs	Plasma, tissue (mouse) and cell lines (human) treated with FABP4 inhibitor	Molecular inhibition of FABP4 activity	Suhre *et al*., J Biomol Screen, 2011
Formate/ acetate in human urine	*NAT2* genotype	Genetic variance in N-acetylase activity	Suhre *et al*., Nature Genetics, 2011
Ratio between phosphorylated and unphosphorylated fibrinogen peptides	*ABO*, *ALPL*, and *FUT2* genotypes	Genetic variance in fibrinogen phosphorylation	Suhre *et al*., Nature, 2011

In all studies pairs of metabolites were identified by a high increase in the strength of association when ratios were used. Note that all of these metabolite pairs are found to be biochemically related to the concrete biological questions of these studies (Interpretation). However, they were singled out from the large number of all possible metabolite pair combinations on the basis of the p-gain without any prior hypotheses.

Several reasons explain why metabolite ratios provide additional information in these association studies: (1) Ratios between related metabolite pairs reduce the overall biological variability in the dataset and thereby increase statistical power. For instance, study participants may have strongly varying nutrition habits, which introduce high variance in the distribution of that nutrient, but also in those of its biochemical break-down products. However, individuals who consume a higher amount of a certain nutrient also exhibit higher levels of its biochemical break-down products. Ratios between these metabolites can thus be considered as some kind of internal normalization. (2) Systematic experimental errors, such as variance in the concentration of external standards result in errors that are comparable for certain metabolite pairs. Such errors are cancelled out in ratios and thereby reduce the overall noise in the dataset. (3) Probably most importantly, when a metabolite pair is connected by a biochemical pathway, metabolite ratios approximate the corresponding reaction rate under idealized steady state assumptions. Metabolite ratios then represent a biologically most relevant entity, namely the flux through a biochemical pathway. For example, in Suhre *et al.*[13], the association of SNP rs174547 at the *FADS1* locus displayed a p-value of p = 2.3 × 10^-21^ and an explained variance of 5.2 % with concentrations of the omega-6 fatty acid 20:4, whereas the p-value of association with ratios between the fatty acids 20:4 and 20:3 was p = 9.987 × 10^-66^ with an explained variance of 15.3 % [13]. The *FADS1* locus encodes a fatty acid delta-5 desaturase. This is a key enzyme in the metabolism of long chain polyunsaturated omega-3 and omega-6 fatty acids. The fatty acids 20:4 and 20:3 are the respective product and substrate pair of the FADS1 reaction [14,11]. The p-gain is defined as the increase in the strength of association, expressed as the change in p-value when using ratios compared to the smaller of the two p-values when using two metabolite concentrations individually. So far, the number of analyzed metabolite concentrations was applied as an ad-hoc critical value for the p-gain. Any association that displayed a p-gain below this number was considered to have occurred by chance. This approach can merely be regarded as an intuitive rule of thumb, since a statistical determination of the distribution of the p-gain and herewith of the critical values has not yet been conducted. In this paper, we derive critical values through determination of the distribution of the p-gain and provide a density table for readout of critical values. In addition, we investigate the characteristics of the p-gain in the situation of Bonferroni correction for multiple tests.

## Results and discussion

### Formal definition of the p-gain

Testing ratios between two metabolite concentrations *a* and *b* should be independent of their order. It is therefore advisable to use log-scaled metabolite ratios in the tests for association. Due to the property log(*a*/*b*) = -log(*b*/*a*) this also halves the multiple testing burden. Moreover, in many of the cases we tested, the distribution of metabolite ratios was observed to be better represented by a log-normal distribution than by a normal distribution. For instance, a test of normality in the study by Illig *et al.*[12] showed that in 85.1 % of the cases, the log-transformed ratio distribution was significantly better represented by a normal distribution than when untransformed ratios were used.

The p-gain was introduced in order to measure whether the association with a genetic locus is significantly stronger for a metabolite ratio than for the belonging metabolite concentrations. As notation, we use ‘p-value(M_1_ | X)’, short ‘P(M_1_)’, to reference the p-value corresponding to a test for association between a trait X (in a GWAS this would be a genetic locus represented by a SNP and in an MWAS it would be a phenotypic trait) and the metabolite M_1_. With this definition, the p-gain for the association of the ratio M_1_/M_2_ of metabolites M_1_ and M_2_ with a trait X is defined as

(1) p‐gain(M1M2|X):=min(p‐value(M1|X),p‐value(M2|X))p‐value(M1M2|X)

### Conservative critical p-gain values for common statistics

Although the p-gain is now frequently used in MWAS and in GWAS with metabolic traits, only a rule of thumb for the determination of critical values has been applied so far. The p-gain was considered as being significant when its value exceeded the number of analyzed metabolite concentrations, that is, the number of additionally performed tests [11-13]. Here we derive critical values of the p-gain by determination of the distribution to define a more sensible threshold. As the distribution of the p-gain depends on the correlation structure among the metabolites, conservative critical values are beneficial in case of analyzing multiple sets of metabolites, since they can be applied to all analyzed settings. For this purpose, we use a universal p-gain defined as the ratio of p-values belonging to two uncorrelated metabolites:

(2) $$\begin{array}{ccc} \text{p‐gain}\left(\frac{M_{1}}{M_{2}}|X\right) & := & \frac{\text{p‐value}(M_{1}|\text{X})}{\text{p‐value}(M_{1}/M_{2}|\text{X})}\text{,}\\ & & \text{cor}(M_{1},M_{1}/M_{2})=0 \end{array}$$

Critical values of the distribution of this p-gain are conservative to the critical values of the distribution of the p-gain given in equation (1), because

$$\text{p‐value}(M_{1}|X)\geq \min(\text{p‐value}(M_{1}|X),\text{p‐value}(M_{2}|X))$$

and therefore

$$\frac{\text{p‐value}(M_{1}|X)}{\text{p‐value}(M_{1}/M_{2}|X)}\geq \frac{\text{min}(\text{p‐value}(M_{1}|X),\text{p‐value}(M_{2}|X))}{\text{p‐value}(M_{1}/M_{2}|X)}$$

The variation of the distribution of the p-gain defined in equation (2) depends on the correlation between M_1_ and M_1_/M_2_. For example, highly correlated metabolic traits contain mainly the same information and have similar p-values in association tests. This results in p-gain values which are close to one. Hence, the variation of the distribution is small. In contrast, weakly correlated metabolic traits contain different information and may have different p-values in association tests. This results in p-gain values distributed broadly around the one. Therefore, assuming $\text{cor}(M_{1},M_{1}/M_{2})=0$, as it was done in equation (2), results in a distribution of the p-gain with largest possible variation and leads to the most conservative critical values.

**Figure 1:**
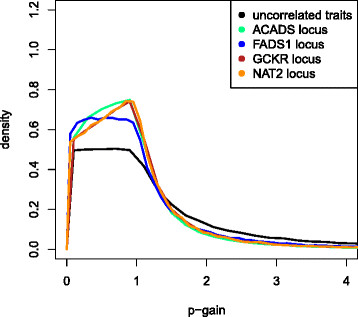
**Distribution of the p-gain.** This Figure shows the distribution of the p-gain for the calculated conservative p-gain of uncorrelated traits as well as for four loci which were significant in Suhre *et al.*[13]. The *ACADS* locus was found to be associated with butyrylcarnitine/propionylcarnitine, *FADS1* with 1-arachidonoylglycerophosphoethanolamine/1-linoleoylglycerophosphoethanolamine, *GCKR* with glucose/mannose and *NAT2* with 1-methylxanthine/4-acetamidobutanoate. The correlations among the metabolite concentrations as well as with the metabolite ratio are summarized in Table S2.

In the situation of the universalized p-gain (equation (2)) we can use the convolution formula for density ratios which yields a split density (see Methods):

(3) $$f_{\frac{P(M_{1})}{P(M_{1}/M_{2})}}\left(p-\text{gain}\right)=\{\begin{array}{cc} \frac{1}{2} & 0<p-\text{gain}<1\\ \frac{1}{2\cdot p-{\text{gain}}^{2}} & p-\text{gain}\geq 1 \end{array}$$

as displayed in Figure 1 (black line). To determine critical values, we derive the cumulative distribution function of the density, i.e.

(4) $$\begin{array}{ccc} F_{\frac{P(M_{1})}{P(M_{1}/M_{2})}}(\text{p‐gain}) & = & \int_{0}^{\text{p‐gain}}f_{\frac{P(M_{1})}{P(M_{1}/M_{2})}}\\ \;(x)dx & = & \left\{ \begin{array}{cc} \frac{1}{2}\text{p‐gain} & 0<\text{p‐gain}<1\\ 1-\frac{1}{2\cdot \text{p‐gain}} & \text{p‐gain}\;\geq \;1 \end{array}\right. \end{array}$$

Herewith, the critical value becomes $\frac{1}{2\cdot \alpha }$ with α denoting the level of significance. In the case of typically used α levels of 0.05, this yields a corresponding critical value for the p-gain of ten. General quantiles are provided in Table S1 (Additional file 1).

### Critical values for multiple testing

In MWAS and in GWAS with metabolomics a large number of ratios are tested in parallel. Therefore, a correction for multiple testing has to be applied. We select Bonferroni correction as the most conservative method. When admitting a type I error rate of α and applying a correction for B tests, i.e. aiming at a level of significance of α/B, the critical value for the p-gain then becomes B/(2⋅α) (see Methods). For example, assumption of a type I error rate of α = 0.05 leads to a critical value of 10⋅Bwhich implies that for Bonferroni correction of B tests the uncorrected critical value of ten can be multiplied by the number of tests B. Hence, the critical value of the p-gain in the situation of multiple testing is not equal to the number of analyzed metabolite concentrations, which was used so far as an ad-hoc criterion, but rather ten times the number of tested ratios.

### P-gain for correlated metabolites

The case of uncorrelated metabolites (equation (2)) is conservative with respect to the p-gain as defined in equation (1). Here we analyze the density of the p-gain as defined in equation (1) for selected correlation settings. In the situation of correlated metabolic traits the convolution formula cannot be applied anymore. Thus, we simulate the density using a copula to generate the correlation among the metabolic traits. A copula is a joint probability distribution whose one-dimensional marginal distributions are uniformly distributed over the interval [0,1]. It takes the dependency among the marginal distributions into account (see Methods). Quantiles for the p-gain densities of correlated metabolic traits are provided in Table S1 (Additional file 1) for various correlation settings. It can be observed that when any of the correlations *cor(M*_*1*_*, ratio)* or *cor(M*_*2*_*, ratio)* increase, the values of the quantiles of the p-gain decrease. This observation can be explained by the fact that the variation of the p-gain can be reduced by increasing the correlation between a metabolite concentration and the ratio (i.e. *cor(M*_*1*_*, ratio)* or *cor(M*_*2*_*, ratio)*). A reduction of the variation of the p-gain leads to smaller critical values. On the other hand, for fixed *cor(M*_*1*_*, ratio)* or *cor(M*_*2*_*, ratio)*, an increase in the correlation between M_1_ and M_2_ leads to an increase in the values for the p-gain quantiles when the correlation between M_1_ and M_2_ is not close to 0. Extending these observations to the most extreme case of having fully correlated metabolite concentrations which are uncorrelated with their ratio (i.e. *cor(M*_*1*_*,M*_*2*_*) = 1, cor(M*_*1*_*, ratio) = 0, cor(M*_*2*_*, ratio) = 0)* we get the largest critical values and thus these critical values are conservative to all correlation settings. This idealized case reduces the p-gain as defined in equation (1) to the p-gain as defined in equation (2). For this case, we derived the distribution using the convolution formula as well as through a simulation. In both cases, the simulated and calculated density as well as the belonging critical values coincided (Table S1, Figure S1 (Additional file 1)). To determine the density of the p-gain for a given correlation setting among the metabolite concentrations and their ratio, the exact distribution of the p-gain for a given metabolite ratio can be simulated using the R-script which is provided as Supplemental Material (Additional file 2).

### Dependence on sample size in real data

In order to examine the behavior of the p-gain in the situation of real data, we compute the observed correlation structure among metabolite ratios which were published in Suhre *et al.*[13] (Table S2). This dataset includes nearly uncorrelated metabolites, such as the ratio between 1-methylxanthine and 4-acetamidobutanoate (association with the *NAT2* locus) as well as highly correlated metabolites, such as the androsterone sulfate to epiandrosterone sulfate ratio (association with the *AKR1C* locus). The distributions of exemplary metabolite ratios are presented in Figure 1. As expected, the densities for correlated metabolic traits display smaller variations than the density for uncorrelated metabolic traits. The observed p-gain values in 1,768 samples of the KORA study vary between 2.79 x 10^3^ and 1.68 x 10^66^ for the 20 loci published in Suhre *et al.* (see Table S3 (Additional file 1)). Using this dataset we conducted simulation tests to address the influence of the sample size on the observed p-gain values. We chose randomly sets of samples sizes between 100 and 2000 samples from the KORA study and calculated the p-gain for these sets. The results of this analysis illustrate the dependence of the p-gain values on the sample size (Table S3 (Additional file 1)). For example, we observe for the association between the *ACADS* locus and the butyrylcarnitine to propionylcarnitine ratio a median p-gain value of 1.4 x 10^2^ for a sample size of N = 100, of 1.1 x 10^5^ for N = 500, of 2.8 x 10^10^ for N = 1000, of 3.1 x 10^15^ for N = 1500 and of 1.4 x 10^21^ for N = 2000.

## Conclusions

We derived critical values for the p-gain to determine significance in various situations. We recommend the use of metabolite ratios and the p-gain statistic when analyzing large scale metabolomics data sets and to apply the critical values with correction of multiple testing as provided in this paper. Given the success of the approach in the metabolomics field, hypothesis free testing of ratios between biologically related quantitative traits should also be considered for association studies with other ‘omics datasets.

## Methods

### Study description

The KORA (Cooperative Health Research in the Region of Augsburg) study is a series of independent population-based epidemiological surveys and follow-up studies of participants living in the region of Augsburg, Southern Germany [15]. All participants are residents of Germany with a German nationality. All participants gave signed informed consent. The study was approved by the local ethics committee (“Bayerische Landesärztekammer”). The present study includes data of the follow-up study KORA F4 (2006-2008) of the KORA S4 survey (1999-2000). For genotyping, we included 1,814 randomly selected participants of KORA F4. The KORA F4 samples were genotyped with the Affymetrix Human SNP Array 6.0 and imputed with IMPUTE v0.4.2 based on Hap Map II [12].

#### Blood collection

We collected blood samples between 2006 and 2008 during the KORA F4 examinations. To avoid variation due to circadian rhythm, blood was drawn in the morning between 8:00 a.m. and 10:00 a.m. after a period of overnight fasting. Blood was drawn into serum gel tubes, gently inverted two times and then allowed to rest for 30 min at room temperature (18 − 25 °C) to obtain complete coagulation. The material was then centrifuged for 10 min and 2,750 *g* at 15 °C. Serum was divided into aliquots and kept for a maximum of 6 h at 4 °C, after which it was deep-frozen to −80 °C until analysis.

#### Metabolomics measurements

On 1,768 fasting serum samples of the KORA F4 study for which we had already genotypes available, metabolic profiling was done using ultrahigh performance liquid-phase chromatography and gas chromatography separation coupled with tandem mass spectrometry [16-18] at Metabolon, an US-based commercial supplier of metabolic analyses. They achieved highly efficient profiling (24 minutes/sample) with low median process variability (<12 %) of more than 250 metabolites, covering over 60 biochemical pathways of human metabolism. A more detailed description of the metabolomics measurement and quality control can be found in Suhre *et al.*[13].

### Statistical analyses

#### Density of p-gain for uncorrelated metabolites (calculation)

The p-gain for two uncorrelated metabolites is defined as:

$$\begin{array}{ccc} p-\text{gain}\left(\frac{M_{1}}{M_{2}}|X\right) & := & \frac{p-\text{value}(M_{1}|\text{X})}{p-\text{value}(M_{1}/M_{2}|\text{X})}\text{,}\\ & & \text{cor}(M_{1},M_{1}/M_{2})=0 \end{array}$$

We calculated the density of the p-gain of two uncorrelated metabolites by using the convolution formula for ratios:

$$\begin{array}{ccc} f_{\frac{P(M_{1})}{P(M_{1}/M_{2})}}(p-\text{gain}) & = & \int_{-\infty }^{+\infty }|t|f_{P(M_{1})}(p-\text{gain}\cdot t)\cdot f_{P(M_{1}/M_{2})}(t)dt\\ & & \forall p-\text{gain}\in R^{+} \end{array}$$

with P(M_1_) and P(M_1_/M_2_) having a uniform distribution on the interval [0,1]. Transformations lead to

fP(M1)P(M1/M2)(p-gain)=∫−∞+∞|t|fP(M1)(p-gain⋅t)⋅fP(M1/M2)(t)dt=∫01t⋅fP(M1)(p-gain⋅t)dt={∫01p-gaintdt=12⋅p-gain2,p-gain≥1∫01tdt=12,0<p-gain<1

The corresponding cumulative distribution is

$$\begin{array}{c} F_{\frac{P(M_{1})}{P(M_{1}/M_{2})}}(p-\text{gain})=\int_{0}^{p-\text{gain}}f_{\frac{P(M_{1})}{P(M_{1}/M_{2})}}(t)dt\\ \;=\left\{ \begin{array}{c} \frac{1}{2}p-\text{gain}\text{,}\;0<p-\text{gain}<1\\ 1-\frac{1}{2\cdot p-\text{gain}}\text{,}\;p-\text{gain}\geq 1 \end{array}\right. \end{array}$$

Therefore,

$$\begin{array}{ccc} F_{\frac{P(M_{1})}{P(M_{1}/M_{2})}}(p-\text{gain}) & = & \left(1-\frac{\alpha }{B}\right)\\ & \Leftrightarrow & 1-\frac{1}{2\cdot p-\text{gain}}=(1-\frac{\alpha }{B})\\ & \Leftrightarrow & p-\text{gain}=\frac{B}{2\alpha }\text{,}\;\text{if}\frac{\alpha }{B}\leq 0.5\text{,} \end{array}$$

with α/Bbeing the significance level α, Bonferroni-corrected for B tests.

#### Density of the p-gain (simulation)

To determine the density of the p-gain we assumed a given correlation structure among the metabolic traits. This confers to a correlation structure among p-values corresponding to these metabolic traits. With these correlated p-values the density of the p-gain can be derived. For simulation of the variables with a given correlation structure we choose the “copula” package [19,20] of the R-Project Environment [21]. The simulated variables were marginal distributions of a multivariate distribution with a uniform distribution on the interval [0,1]. We then transformed the simulated variables with an inverse normal transformation to gain a normal distribution which is essential for linear regressions. To simulate the p-values belonging to these variables, we generated additional variables and conducted linear regressions where these additional variables were the independent and the variables simulated with the copula the dependent variables. The received p-values contain a correlation structure which belongs to the correlation structure of the metabolic traits. Out of these p-values, we calculated a density of the p-gain empirically and derived critical values for given significance levels. An R-script with the simulation commands is provided with the supplemental material.

#### Dependence of p-gain values on sample size

We determined the dependency of the p-gain of the sample size by drawing randomly between 100 and 2000 samples from the KORA data (with replacement). For each sample size, we repeated this analysis 1500 times. For all sample subsets we calculated the p-gain. We then determined the median p-gain as well as the 1^st^ and 3^rd^ quantile of the p-gains for each sample size.

## Competing interests

The authors declare that they have no competing interests.

## Authors' contributions

AKP designed the study, performed the statistical analysis and wrote the manuscript. JK provided data and critically reviewed the manuscript. BW and FJT provided data. HEW provided material. CG and KS designed the study and critically reviewed the manuscript. All authors read and approved the final manuscript.

## Supplementary Material

Additional file 1 **Supplementary Figure S1 and Tables S1-S3.**This file contains supplementary information. Click here for file

Additional file 2 **R-script for simulation of the distribution of the p-gain.**This file contains supplementary information. Click here for file
