# Supplementary material for: On the hypothesis-free testing of metabolite ratios in genome-wide and metabolome-wide association studies
Source: BMC Bioinformatics. 2012 Jun 6;13:120. doi: 10.1186/1471-2105-13-120 (PMC3537592; doi:10.1186/1471-2105-13-120)
Supplement: Additional file 1 — Supplementary Figure S1 and Tables S1-S3.This file contains supplementary information. [file 1471-2105-13-120-S1.pdf]

Additional file 1

**Item**	**Description**
Figure S1	Calculated and simulated distribution for p-gain of uncorrelated metabolic traits
Table S1	Quantiles of the p-gain density
Table S2	Correlation among metabolite ratios published by Suhre *et al.*
Table S3	P-gain values of metabolon ratios published by Suhre *et al.* for various sample sizes

Figure S1 – Calculated and simulated distribution for p-gain of uncorrelated metabolic traits

This Figure shows that the calculated distribution of the p-gain for uncorrelated metabolic traits and the simulated distribution coincide. On the x-axis is the p-gain entered and on the y-axis the density. The red line is the calculated density whereas the black line is the simulated density.

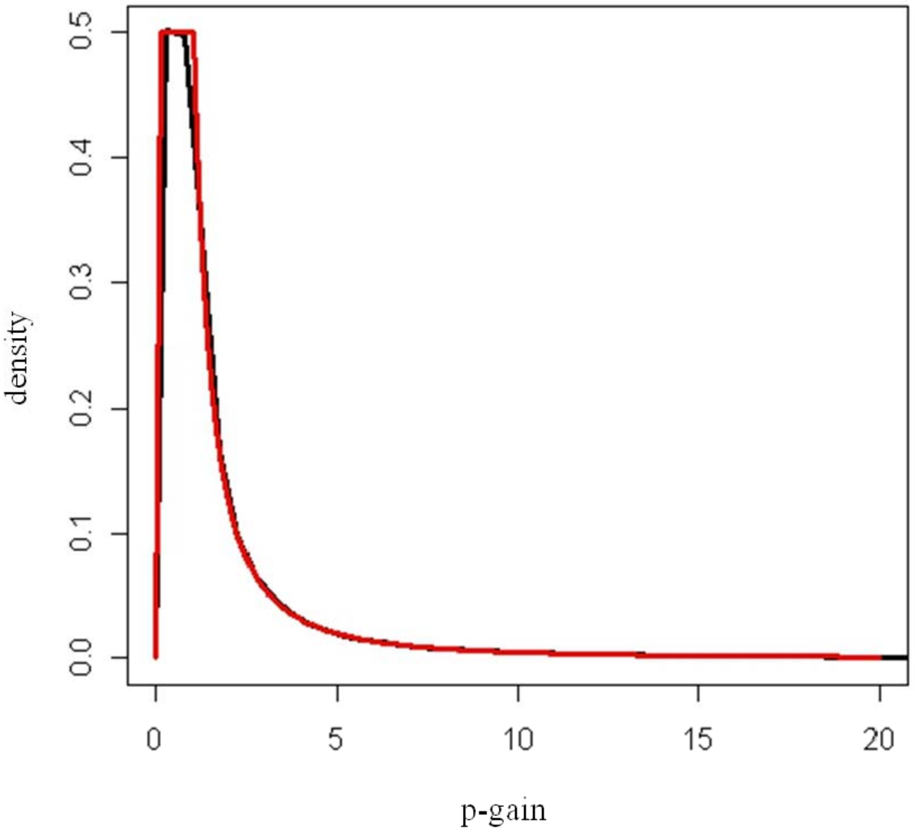

Table S1 – Quantiles of the p-gain density

Reported are the quantiles for various combinations of correlation values among the metabolites M_1 and M_2 as well as their ratio M_1/M_2. For the case of uncorrelated metabolic traits, the quantiles for the simulated (sim) and theoretic (theo) densities are specified.

Correlation			Quantiles								
**M_1; M_2**	**M_1; ratio**	**M_2; ratio**	**0.01**	**0.025**	**0.05**	**0.1**	**0.5**	**0.9**	**0.95**	**0.975**	**0.99**
0	0	0	34.13	13.18	6.59	3.30	0.63	0.10	0.05	0.02	0.01
0	0	±0.2	32.25	12.45	6.48	3.25	0.64	0.11	0.05	0.03	0.01
0	0	±0.4	25.57	10.76	5.80	3.04	0.64	0.11	0.06	0.03	0.01
0	0	±0.6	17.82	8.28	4.71	2.69	0.65	0.13	0.07	0.04	0.02
0	0	±0.8	8.54	4.97	3.25	2.10	0.67	0.16	0.09	0.05	0.02
0	0	±1.0	1.00	1.00	1.00	1.00	0.99	0.20	0.10	0.05	0.02
0	±0.2	±0.2	28.46	11.86	6.21	3.15	0.64	0.11	0.05	0.03	0.01
0	±0.2	±0.4	23.96	10.44	5.61	2.97	0.64	0.12	0.06	0.03	0.01
0	±0.2	±0.6	15.63	7.64	4.48	2.57	0.65	0.13	0.07	0.04	0.02
0	±0.2	±0.8	7.83	4.61	3.04	2.01	0.67	0.16	0.09	0.05	0.02
0	±0.4	±0.4	18.27	8.44	4.78	2.70	0.64	0.12	0.07	0.03	0.01
0	±0.4	±0.6	12.15	6.42	3.82	2.32	0.65	0.14	0.08	0.04	0.02
0	±0.4	±0.8	5.82	3.61	2.53	1.78	0.68	0.18	0.10	0.06	0.03
0	±0.6	±0.6	7.80	4.50	2.98	1.94	0.66	0.16	0.09	0.06	0.03
0	±0.6	±0.8	3.27	2.26	1.73	1.34	0.72	0.21	0.13	0.8	0.04
0	±0.8	±0.8	3.16	2.23	1.72	1.34	00.71	0.22	0.13	0.08	0.04
±0.2	0	0	32.68	13.20	6.53	3.30	0.64	0.10	0.05	0.02	0.01
±0.2	0	±0.2	31.44	12.59	6.50	3.27	0.64	0.11	0.05	0.03	0.01
±0.2	0	±0.4	25.23	11.19	5.81	3.07	0.64	0.12	0.06	0.03	0.01
±0.2	0	±0.6	16.49	8.05	4.62	2.65	0.65	0.13	0.07	0.04	0.01
±0.2	0	±0.8	8.64	4.92	3.24	2.11	0.68	0.16	0.09	0.05	0.02
±0.2	±0.2	±0.2	29.32	12.72	6.35	3.23	0.64	0.11	0.05	0.03	0.01
±0.2	±0.2	±0.4	24.63	10.72	5.61	2.96	0.64	0.12	0.06	0.03	0.01
±0.2	±0.2	±0.6	16.02	7.95	4.61	2.64	0.65	0.13	0.07	0.04	0.02
±0.2	±0.2	±0.8	8.25	4.82	3.15	2.08	0.67	0.16	0.09	0.05	0.02

±0.2	±0.2	±1.0	1.00	1.00	1.00	1.00	0.99	0.21	0.11	0.06	0.02
±0.2	±0.4	±0.4	21.08	9.46	5.20	2.84	0.65	0.12	0.07	0.03	0.02
±0.2	±0.4	±0.6	14.16	7.00	4.17	2.45	0.65	0.14	0.08	0.04	0.02
±0.2	±0.4	±0.8	7.19	4.24	2.86	1.93	0.67	0.17	0.10	0.05	0.02
±0.2	±0.6	±0.6	9.65	5.22	3.34	2.12	0.66	0.16	0.09	0.05	0.03
±0.2	±0.6	±0.8	4.66	3.01	2.19	1.60	0.67	0.20	0.12	0.07	0.04
±0.2	±0.8	±0.8	2.48	1.85	1.49	1.22	0.71	0.25	0.16	0.11	0.06
±0.4	0	0	33.47	13.62	6.85	3.42	0.65	0.11	0.05	0.03	0.01
±0.4	0	±0.2	32.34	12.73	6.72	3.38	0.66	0.11	0.06	0.03	0.01
±0.4	0	±0.4	25.58	11.09	5.90	3.13	0.66	0.12	0.06	0.03	0.02
±0.4	0	±0.6	17.22	8.18	4.66	2.69	0.68	0.13	0.07	0.04	0.02
±0.4	0	±0.8	7.77	4.58	3.05	2.04	0.71	0.17	0.09	0.05	0.02
±0.4	±0.2	±0.2	29.70	12.35	6.41	3.30	0.66	0.11	0.06	0.03	0.01
±0.4	±0.2	±0.4	25.85	11.19	5.92	3.10	0.66	0.12	0.06	0.03	0.01
±0.4	±0.2	±0.6	18.47	8.57	4.87	2.75	0.67	0.14	0.07	0.04	0.02
±0.4	±0.2	±0.8	8.76	5.01	3.28	2.15	0.70	0.17	0.09	0.05	0.02
±0.4	±0.4	±0.4	21.56	9.93	5.38	2.91	0.66	0.13	0.07	0.04	0.01
±0.4	±0.4	±0.6	14.84	7.62	4.38	2.58	0.67	0.14	0.08	0.04	0.02
±0.4	±0.4	±0.8	8.46	4.84	3.16	2.07	0.69	0.18	0.10	0.06	0.03
±0.4	±0.4	±1.0	1.00	1.00	1.00	1.00	1.00	0.23	0.12	0.07	0.03
±0.4	±0.6	±0.6	11.44	6.04	3.72	2.28	0.66	0.16	0.09	0.05	0.03
±0.4	±0.6	±0.8	6.16	3.80	2.61	1.81	0.68	0.20	0.12	0.07	0.04
±0.4	±0.8	±0.8	3.12	2.24	1.75	1.37	0.69	0.25	0.17	0.11	0.07
±0.6	0	0	35.09	14.02	7.15	3.54	0.70	0.12	0.06	0.03	0.01
±0.6	0	±0.2	32.62	13.16	6.79	3.45	0.70	0.12	0.06	0.03	0.01
±0.6	0	±0.4	25.85	11.29	5.97	3.17	0.70	0.13	0.06	0.03	0.01
±0.6	0	±0.6	15.78	7.78	4.64	2.68	0.73	0.15	0.08	0.04	0.02
±0.6	0	±0.8	6.39	3.90	2.69	1.88	0.80	0.18	0.09	0.05	0.02
±0.6	±0.2	±0.2	31.86	13.11	6.83	3.46	0.69	0.12	0.06	0.03	0.01
±0.6	±0.2	±0.4	27.85	11.48	6.14	3.23	0.70	0.13	0.07	0.03	0.01
±0.6	±0.2	±0.6	17.86	8.71	5.01	2.86	0.71	0.15	0.08	0.04	0.02
±0.6	±0.2	±0.8	8.27	4.83	3.24	2.15	0.75	0.18	0.09	0.05	0.02
±0.6	±0.4	±0.4	25.18	10.85	5.74	3.13	0.70	0.13	0.07	0.04	0.02

±0.6	±0.4	±0.6	16.80	8.23	4.79	2.73	0.70	0.15	0.08	0.04	0.02
±0.6	±0.4	±0.8	8.76	5.14	3.36	2.20	0.73	0.18	0.10	0.06	0.02
±0.6	±0.6	±0.6	13.19	6.79	4.18	2.51	0.70	0.16	0.09	0.05	0.03
±0.6	±0.6	±0.8	7.54	4.51	3.00	2.01	0.71	0.20	0.12	0.07	0.04
±0.6	±0.6	±1.0	1.00	1.00	1.00	1.00	1.00	0.27	0.16	0.09	0.04
±0.6	±0.8	±0.8	4.50	3.01	2.23	1.63	0.71	0.25	0.17	0.11	0.07
±0.8	0	0	38.42	15.56	7.75	3.92	0.76	0.13	0.06	0.03	0.01
±0.8	0	±0.2	35.55	14.04	7.21	3.70	0.76	0.13	0.07	0.03	0.01
±0.8	0	±0.4	25.56	11.38	6.19	3.34	0.79	0.15	0.07	0.04	0.01
±0.8	0	±0.6	14.46	7.00	4.26	2.59	0.84	0.17	0.09	0.04	0.02
±0.8	0	±0.8	9.38	5.33	3.41	2.21	0.82	0.18	0.09	0.05	0.02
±0.8	±0.2	±0.2	34.37	14.11	7.24	3.74	0.76	0.14	0.07	0.03	0.01
±0.8	±0.2	±0.4	30.08	12.81	6.75	3.55	0.77	0.14	0.07	0.03	0.01
±0.8	±0.2	±0.6	18.04	8.76	5.10	2.98	0.80	0.17	0.09	0.04	0.02
±0.8	±0.2	±0.8	8.55	4.99	3.32	2.20	0.84	0.20	0.10	0.05	0.02
±0.8	±0.4	±0.4	26.61	11.53	6.23	3.37	0.76	0.15	0.08	0.04	0.02
±0.8	±0.4	±0.6	18.11	8.94	5.19	2.98	0.77	0.17	0.09	0.05	0.02
±0.8	±0.4	±0.8	9.32	5.31	3.51	2.32	0.81	0.21	0.11	0.06	0.03
±0.8	±0.6	±0.6	15.78	7.78	4.66	2.77	0.76	0.18	0.10	0.06	0.03
±0.8	±0.6	±0.8	8.98	5.27	3.46	2.25	0.78	0.22	0.13	0.08	0.04
±0.8	±0.8	±0.8	6.24	3.87	2.71	1.92	0.76	0.26	0.17	0.12	0.07
±0.8	±0.8	±1.0	1.00	1.00	1.00	1.00	1.00	0.37	0.24	0.17	0.10
±1.0 (sim)	0 (sim)	0 (sim)	50.72	20.04	10.00	4.99	1.00	0.20	0.10	0.05	0.02
±1.0 (theo)	0 (theo)	0 (theo)	50	20	10	5	1	0.2	0.1	0.05	0.02
±1.0	±0.2	±0.2	47.25	18.75	9.49	4.80	1.00	0.20	0.10	0.05	0.02
±1.0	±0.4	±0.4	38.57	16.16	8.45	4.42	1.00	0.23	0.12	0.06	0.03
±1.0	±0.6	±0.6	22.51	11.12	6.46	3.68	1.00	0.27	0.16	0.09	0.05
±1.0	±0.8	±0.8	9.30	5.65	3.83	2.60	1.00	0.38	0.26	0.18	0.11
±1.0	±1.0	±1.0	1.00	1.00	1.00	1.00	1.00	1.00	1.00	1.00	1.00

Table S2 – Correlation among metabolite ratios published by Suhre *et al.*

This Table summarizes the correlation structure among the 20 metabolite ratios which were published by Suhre *et al.* [13]. Metabolite M_1 corresponds to the numerator of the metabolite ratios and metabolite M_2 to the denominator.

**Label**	**Metabolite ratio**	**Correlation**		
		**M_1; M_2**	**M_1; ratio**	**M_2; ratio**
ACADS	butyrylcarnitine / propionylcarnitine	0.422	0.769	-0.255
FADS1	1-arachidonoylglycerophospho-ethanolamine/1-linoleoylglycero-phosphoethanolamine	0.615	-0.547	0.323
UGT1A	bilirubin (E,E)/oleoylcarnitine	0.627	0.731	-0.073
ACADM	hexanoylcarnitine/oleate (18:1n9)	0.498	0.777	-0.159
SCD	myristate (14:0)/myristoleate (14:1n5)	0.830	-0.131	-0.662
GCKR	glucose/mannose	0.589	0.012	-0.801
NAT2	1-methylxanthine/ 4-acetamidobutanoate	0.038	0.896	-0.410
ABO	ADpSGEGDFXAEGGGVR/ ADSGEGDFXAEGGGVR	0.407	0.724	-0.335
CYP4A	10-nonadecenoate (19:1n9)/ 10-undecenoate (11:1n1)	0.555	0.555	-0.383
SLCO1B1	eicosenoate (20:1n9 or 11)/ tetradecanedioate	0.303	0.513	-0.662
FUT2	ADpSGEGDFXAEGGGVR/ ADSGEGDFXAEGGGVR	0.407	0.724	-0.335
ENPEP	ADpSGEGDFXAEGGGVR/ DSGEGDFXAEGGGVR	0.511	0.393	-0.589
AKR1C	androsterone sulfate/ epiandrosterone sulfate	0.920	0.464	0.081
ALPL	ADpSGEGDFXAEGGGVR/ DSGEGDFXAEGGGVR	0.511	0.393	-0.589
SLC7A6	glutaroyl carnitine/lysine	0.011	0.862	-0.497
PDXDC1	1-eicosatrienoylglycerophospho-choline/1-linoleoylglycerophosph o-choline	0.579	0.676	-0.210
AHR	caffeine / quinate	0.207	0.748	-0.495
ELOVL2	docosahexaenoate (DHA; 22:6n3)/ eicosapentaenoate (EPA; 20:5n3)	0.771	0.203	-0.467
IVD	3-(4-hydroxyphenyl)lactate/ isovalerylcarnitine	0.327	0.552	-0.607
SLC16A10	isoleucine/tyrosine	0.441	0.592	-0.462

Table S3 – P-gain values of metabolon ratios published by Suhre *et al*. for various sample sizes

This Table shows the dependence of the p-gain on the sample sizes for 20 significant metabolite ratios published by Suhre *et al*. [13]. The label of the locus, the metabolite ratio, SNP, MAF (%) and observed p-gain in the 1768 samples of the KORA study are given for each locus. Furthermore, the median as well as 1st and 3rd quartiles are specified for randomly drawn sample subsets from the KORA study.

Label	Metabolite ratio	SNP	MAF (%)	p-gain (N=1768)	sample sizes N=100	N=500	N=1000	N=1500	N=2000
ACADS	butyrylcarnitine / propionylcarnitine	rs2066938	25.2	6.15×10^{42}	$1.4x10^{2}$ ($3.0x10^{0}$; $6.4x10^{3}$)	$1.1x10^{5}$ ($8.3x10^{1}$; $1.7x10^{11}$)	$2.8x10^{10}$ ($2.1x10^{3}$; $4.9x10^{18}$)	$3.1x10^{15}$ ($8.6x10^{4}$; $2.7x10^{27}$)	$1.4x10^{21}$ ($2.5x10^{7}$; $2.0x10^{36}$)
FADS1	1-arachidonoyl-glycerophospho-ethanolamine/1-linoleoylglycero-phosphoethanolamine	rs174547	32.0	1.68×10^{66}	$1.4x10^{3}$ ($1.2x10^{2}$; $2.2x10^{4}$)	$3.1x10^{8}$ ($1.4x10^{3}$; $4.1x10^{17}$)	$4.1x10^{17}$ ($2.2x10^{4}$; $1.2x10^{32}$)	$4.8x10^{25}$ ($3.2x10^{8}$; $3.3x10^{44}$)	$2.7x10^{35}$ ($1.6x10^{15}$; $2.2x10^{58}$)
UGT1A	bilirubin (E,E)/ oleoylcarnitine	rs887829	33.7	2.63×10^{32}	$4.1x10^{1}$ ($7.3x10^{0}$; $3.3x10^{2}$)	$3.2x10^{4}$ ($4.1x10^{1}$; $5.6x10^{8}$)	$4.9x10^{8}$ ($3.2x10^{2}$; $9.7x10^{14}$)	$3.1x10^{12}$ ($3.2x10^{4}$; $4.3x10^{21}$)	$2.1x10^{17}$ ($1.1x10^{7}$; $2.7x10^{28}$)
ACADM	hexanoylcarnitine/ oleate (18:1n9)	rs211718	30.4	1.91×10^{10}	$3.7x10^{0}$ ($7.4x10^{-1}$; $1.7x10^{1}$)	$1.8x10^{1}$ ($1.5x10^{0}$; $1.2x10^{3}$)	$2.5x10^{2}$ ($3.9x10^{0}$; $2.0x10^{5}$)	$6.1x10^{3}$ ($1.0x10^{1}$; $2.4x10^{7}$)	$1.3x10^{5}$ ($3.2x10^{1}$; $3.4x10^{9}$)
SCD	myristate (14:0)/ myristoleate (14:1n5)	rs603424	18.9	4.22×10^{35}	$5.1x10^{1}$ ($9.5x10^{0}$; $4.7x10^{2}$)	$7.1x10^{4}$ ($5.1x10^{1}$; $4.1x10^{9}$)	$3.5x10^{9}$ ($4.6x10^{2}$; $1.1x10^{17}$)	$8.4x10^{13}$ ($7.2x10^{4}$; $9.1x10^{23}$)	$2.7x10^{19}$ ($8.2x10^{7}$; $5.3x10^{31}$)
GCKR	glucose/mannose	rs780094	39.9	4.22×10^{7}	$4.1x10^{0}$ ($1.1x10^{0}$; $2.7x10^{1}$)	$1.6x10^{1}$ ($1.8x10^{0}$; $4.0x10^{2}$)	$9.5x10^{1}$ ($3.9x10^{0}$; $1.8x10^{4}$)	$7.8x10^{2}$ ($8.4x10^{0}$; $8.6x10^{5}$)	$7.3x10^{3}$ ($2.1x10^{1}$; $2.6x10^{7}$)
NAT2	1-methylxanthine/ 4-acetamidobutanoate	rs1495743	18.8	1.79×10^{9}	$1.7x10^{0}$ ($6.1x10^{-1}$; $4.2x10^{0}$)	$7.6x10^{0}$ ($1.4x10^{0}$; $1.8x10^{2}$)	$1.3x10^{2}$ ($2.8x10^{0}$; $1.5x10^{4}$)	$2.3x10^{3}$ ($7.2x10^{0}$; $1.5x10^{6}$)	$5.0x10^{4}$ ($3.1x10^{1}$; $1.5x10^{8}$)
ABO	ADpSGEGDFXAEG-GGVR/ADSGEGDF-XAEGGGVR	rs612169	33.5	5.06×10^{15}	$3.2x10^{0}$ ($1.1x10^{0}$; $1.0x10^{1}$)	$4.2x10^{1}$ ($3.0x10^{0}$; $4.9x10^{3}$)	$3.8x10^{3}$ ($8.6x10^{0}$; $5.6x10^{6}$)	$3.2x10^{5}$ ($4.1x10^{1}$; $8.7x10^{9}$)	$5.5x10^{7}$ ($5.0x10^{2}$; $1.1x10^{13}$)
CYP4A	10-nonadecenoate (19:1n9)/10-undeceno-ate (11:1n1)	rs9332998	13.5	1.26×10^{9}	$1.9x10^{0}$ ($5.2x10^{-1}$; $7.7x10^{0}$)	$7.0x10^{0}$ ($9.3x10^{-1}$; $1.6x10^{2}$)	$3.8x10^{1}$ ($1.8x10^{0}$; $4.6x10^{3}$)	$2.9x10^{2}$ ($3.5x10^{0}$; $1.7x10^{5}$)	$2.6x10^{3}$ ($7.8x10^{0}$; $6.0x10^{6}$)
SLCO1B1	eicosenoate (20:1n9 or 11)/ tetradecanedioate	rs4149081	20.5	9.63×10^{4}	$1.3x10^{0}$ ($5.1x10^{-1}$; $4.2x10^{0}$)	$4.0x10^{0}$ ($8.4x10^{-1}$; $4.1x10^{1}$)	$1.5x10^{1}$ ($1.4x10^{0}$; $4.6x10^{2}$)	$6.8x10^{1}$ ($2.6x10^{0}$; $5.4x10^{3}$)	$3.3x10^{2}$ ($5.0x10^{0}$; $6.3x10^{4}$)
FUT2	ADpSGEGDFXAEG-GGVR/ADSGEGDF-XAEGGGVR	rs503279	46.4	1.46×10^{5}	$1.2x10^{0}$ ($6.0x10^{-1}$; $2.9x10^{0}$)	$2.9x10^{0}$ ($8.9x10^{-1}$; $2.0x10^{1}$)	$1.0x10^{1}$ ($1.4x10^{0}$; $2.0x10^{2}$)	$4.4x10^{1}$ ($2.3x10^{0}$; $1.8x10^{3}$)	$1.9x10^{2}$ ($4.3x10^{0}$; $1.8x10^{4}$)
ENPEP	ADpSGEGDFXAEG-GGVR/DSGEGDFX-AEGGGVR	rs2087160	20.7	6.22×10^{3}	$1.0x10^{0}$ ($4.6x10^{-1}$; $2.4x10^{0}$)	$1.9x10^{0}$ ($7.0x10^{-1}$; $8.8x10^{0}$)	$4.3x10^{0}$ ($9.4x10^{-1}$; $3.8x10^{1}$)	$1.0x10^{1}$ ($1.4x10^{0}$; $2.0x10^{2}$)	$2.6x10^{1}$ ($1.9x10^{0}$; $9.1x10^{2}$)
AKR1C	androsterone sulfate/ epiandrosterone sulfate	rs2518049	17.5	4.62×10^{4}	$1.3x10^{0}$ ($5.5x10^{-1}$; $4.1x10^{0}$)	$3.0x10^{0}$ ($8.8x10^{-1}$; $1.9x10^{1}$)	$8.3x10^{0}$ ($1.3x10^{0}$; $1.3x10^{2}$)	$2.8x10^{1}$ ($2.2x10^{0}$; $8.1x10^{2}$)	$8.7x10^{1}$ ($3.4x10^{0}$; $4.8x10^{3}$)
ALPL	ADpSGEGDFXAEG-GGVR/DSGEGDFX-AEGGGVR	rs10799701	43.5	8.59×10^{9}	$1.5x10^{0}$ ($5.6x10^{-1}$; $4.5x10^{0}$)	$4.6x10^{0}$ ($9.2x10^{-1}$; $5.4x10^{1}$)	$2.4x10^{1}$ ($1.7x10^{0}$; $1.4x10^{3}$)	$1.7x10^{2}$ ($3.1x10^{0}$; $3.6x10^{4}$)	$1.3x10^{3}$ ($6.7x10^{0}$; $1.3x10^{6}$)
SLC7A6	glutaroyl carnitine/ lysine	rs6499165	26.6	4.29×10^{3}	$1.0x10^{0}$ ($5.2x10^{-1}$; $2.0x10^{0}$)	$2.1x10^{0}$ ($7.5x10^{-1}$; $9.8x10^{0}$)	$5.6x10^{0}$ ($1.1x10^{0}$; $6.9x10^{1}$)	$1.8x10^{1}$ ($1.7x10^{0}$; $4.7x10^{2}$)	$6.8x10^{1}$ ($2.7x10^{0}$; $3.5x10^{3}$)
PDXDC1	1-eicosatrienoylglycero	rs7200543	30.4	5.86×10^{3}	$1.3x10^{0}$	$2.8x10^{0}$	$6.8x10^{0}$	$1.9x10^{1}$	$5.9x10^{1}$

	phosphocholine/1-lino-leoylglycerophospho-choline				(5.7×10^{-1}; 4.0×10^{0})	(7.6×10^{-1}; 1.8×10^{1})	(1.1×10^{0}; 1.1×10^{2})	(1.7×10^{0}; 7.1×10^{2})	(2.7×10^{0}; 4.2×10^{3})
AHR	caffeine / quinate	rs12670403	48.7	1.80×10^{4}	1.0×10^{0} (4.6×10^{-1}; 2.6×10^{0})	2.3×10^{0} (6.5×10^{-1}; 1.7×10^{1})	5.7×10^{0} (9.0×10^{-1}; 1.0×10^{2})	1.8×10^{1} (1.3×10^{0}; 6.3×10^{2})	5.4×10^{1} (2.0×10^{0}; 5.1×10^{3})
ELOVL2	docosahexaenoate (DHA; 22:6n3)/ eicosapentaenoate (EPA; 20:5n3)	rs9393903	24.2	2.26×10^{8}	1.6×10^{0} (6.7×10^{-1}; 5.4×10^{0})	7.7×10^{0} (1.3×10^{0}; 1.4×10^{2})	7.6×10^{1} (2.9×10^{0}; 6.2×10^{3})	8.6×10^{2} (6.9×10^{0}; 3.3×10^{5})	1.1×10^{4} (2.0×10^{1}; 1.4×10^{7})
IVD	3-(4-hydroxyphenyl) lactate/isovaleryl-carnitine	rs10518693	39.6	2.79×10^{3}	1.0×10^{0} (5.3×10^{-1}; 2.3×10^{0})	2.1×10^{0} (7.1×10^{-1}; 1.3×10^{1})	5.5×10^{0} (9.5×10^{-1}; 7.4×10^{1})	1.6×10^{1} (1.3×10^{0}; 5.6×10^{2})	4.9×10^{1} (2.1×10^{0}; 3.1×10^{3})
SLC16A10	isoleucine/tyrosine	rs7760535	40.1	4.84×10^{5}	1.2×10^{0} (5.8×10^{-1}; 2.8×10^{0})	3.3×10^{0} (9.3×10^{-1}; 2.7×10^{1})	1.5×10^{1} (1.5×10^{0}; 2.9×10^{2})	6.9×10^{1} (2.8×10^{0}; 4.5×10^{3})	3.9×10^{2} (6.0×10^{0}; 7.4×10^{4})
