# Supplementary material for: On the hypothesis-free testing of metabolite ratios in genome-wide and metabolome-wide association studies
Source: BMC Bioinformatics. 2012 Jun 6;13:120. doi: 10.1186/1471-2105-13-120 (PMC3537592; doi:10.1186/1471-2105-13-120)
Supplement: Additional file 2 — R-script for simulation of the distribution of the p-gain.This file contains supplementary information. [file 1471-2105-13-120-S2.pdf]

Additional file 2

This R-script simulates the density of the p-gain for metabolite concentrations M_1 and M_2.

Input variables:

`kor_M1_M2`	correlation of M_1 and M_2
`kor_M1_ratio`	correlation of M^1 and the metabolite ratio M_1/M_2
`kor_M2_ratio`	correlation of M^2 and the metabolite ratio M_1/M_2
`alpha`	significance level

Function:

```
p.gain.simulation<-function(kor_M1_M2, kor_M1_ratio, kor_M2_ratio,
alpha)
{
      library(copula)
      korrel<-c(kor_M1_M2, kor_M1_ratio, kor_M2_ratio)
      norm.cop<-normalCopula(korrel^3, dim=3, dispstr="un")

      pval1.l<-pval2.l<-pval3.l<-NULL
      for(j in 1:100)
      {
        test<-rcopula(norm.cop, 5000)
        IVNTR1<- qnorm((rank(test[,1],na.last="keep")-
           0.5)/sum(!is.na(test[,1])))
        IVNTR2<- qnorm((rank(test[,2],na.last="keep")-
           0.5)/sum(!is.na(test[,2])))
        IVNTR3<- qnorm((rank(test[,3],na.last="keep")-
           0.5)/sum(!is.na(test[,3])))

        pval1<-pval2<-pval3<-rep(NA, 1000)
        for(i in 1:1000)
        {
          var1<-runif(5000,min=0, max=1)
          pval1[i]<-summary(lm(IVNTR1~var1))$coefficients[2,4]
          pval2[i]<-summary(lm(IVNTR2~var1))$coefficients[2,4]
          pval3[i]<-summary(lm(IVNTR3~var1))$coefficients[2,4]
        }
        pval1.l<-c(pval1.l, pval1)
        pval2.l<-c(pval2.l, pval2)
        pval3.l<-c(pval3.l, pval3)
      }

      test2<-cbind(apply(cbind(pval1.l, pval2.l),1,min), pval3.l)
      pgain<-test2[,1]/test2[,2]
      pgain.order<-pgain[order(pgain, decreasing=TRUE)]
      print(pgain.order[length(pgain)*alpha])
}
```
